# General practitioners' attitude towards cooperation with other health professionals in managing patients with multimorbidity and polypharmacy: A cross-sectional study

**DOI:** 10.1080/13814788.2022.2044781

**Published:** 2022-05-20

**Authors:** Hélène Carrier, Anna Zaytseva, Aurélie Bocquier, Patrick Villani, Martin Fortin, Pierre Verger

**Affiliations:** a Department of General Practice, Aix Marseille University, Marseille, France; b ORS Paca, Regional Health Observatory, Provence- Alpes-Côte d'Azur, Marseille, France; c Aix Marseille University, CNRS (French National Centre for Scientific Research), EHESS (School of Advanced Studies in the Social Sciences), Centrale Marseille, AMSE (Aix-Marseille School of Economics), France; d Internal Medicine, Geriatrics and Therapeutic Unit, Assistance Publique des Hôpitaux de Marseille (AP-HM), Marseille, France; e Anthropology Bio-Cultural, Law and Ethics (ADES), French Blood Agency (EFS), National Center for Scientific Research (CNRS), Aix Marseille University, Marseille, France; f Department of Family Medicine and Emergency Medicine, Faculty of Medicine and Health Sciences, Université de Sherbrooke, Québec, Canada; g Centre Universitaire de Santé et de Services Sociaux du Saguenay-Lac St-Jean, Chicoutimi, Quebec, Canada

**Keywords:** Multimorbidity, polypharmacy, interprofessional relations, nurse practitioners, pharmacists

## Abstract

**Background:**

Cooperation between general practitioners (GPs) and other healthcare professionals appears to help reduce the risk of polypharmacy-related adverse events in patients with multimorbidity.

**Objectives:**

To investigate GPs profiles according to their opinions and attitudes about interprofessional cooperation and to study the association between these profiles and GPs’ characteristics.

**Methods:**

Between May and July 2016, we conducted a cross-sectional survey of a panel of French GPs about their management of patients with multimorbidity and polypharmacy, focussing on their opinions on the roles of healthcare professionals and interprofessional cooperation. We used agglomerative hierarchical cluster analysis to identify GPs profiles, then multivariable logistic regression models to study their associations with the characteristics of these doctors.

**Results:**

1183 GPs responded to the questionnaire. We identified four profiles of GPs according to their declared attitudes towards cooperation: GPs in the ‘very favourable’ profile (14%) were willing to cooperate with various health professionals, including the delegation of some prescribing tasks to pharmacists; GPs in the ‘moderately favourable’ profile (47%) had favourable views on the roles of health professionals, with the exception for this specific delegation of the task; GPs from the ‘selectively favourable’ profile (27%) tended to work only with doctors; GPs from the ‘non-cooperative’ profile (12%) did not seem to be interested in cooperation. Some profiles were associated with GPs’ ages or participation in continuing medical education.

**Conclusion:**

Our study highlights disparities between GPs regarding cooperation with other professionals caring for their patients and suggests ways to improve cooperation.

KEY MESSAGES

- There are different profiles of GPs regarding their attitudes towards cooperation and sharing of prescriptions management with specialists and other health professionals for patients with multimorbidity and polypharmacy.
- While most GPs recognise the pharmacists’ knowledge about medicines, only a minority would agree to share prescription management with them.

## Introduction

Multimorbidity, defined as the coexistence of two or more diseases in the same individual [1], leads to polypharmacy associated with several iatrogenic risks – including drug interactions and adverse drug reactions – and premature mortality [2]. Multimorbidity also increases the use of primary and secondary health care services [3], making it difficult for general practitioners (GPs) to coordinate care [4]. The number of specialists involved in managing patients with multimorbidity and their prescriptions also increases the risk of adverse drug events [2]. Multimorbidity guidelines recommend regular review of patients' prescriptions, using validated tools such as STOP-START or PIM lists [5]. Interprofessional management of polypharmacy involving case managers, nurse practitioners, or pharmacists, can reduce potentially inappropriate prescribing [6,7], particularly when it involves prescription review by pharmacists [8,9]. Effective cooperation between GPs and other health professionals can also improve the functional status of these patients, the adherence of professionals to recommended practices, and the quality of GPs' practices (e.g. monitoring diabetic patients or prescribing benzodiazepine according to guidelines) [10].

As a result, health policies in several countries encourage interprofessional cooperation and multi-professional practices (e.g. multidisciplinary or multi-professional group practices and healthcare networks) [11,12]. In France, primary care is mainly provided by independent professionals in private practice. Most often, these health professionals (GPs, nurses, physiotherapists, pharmacists, etc.) are installed separately, financially autonomous and cooperate in the form of informal networks. Over the last fifteen years, the title of ‘team health action’ nurses has been created to participate in the follow-up of patients with chronic diseases, alongside GPs with whom they sign a collaboration protocol. The primary role of these nurses is the therapeutic education of patients. In recent years, several medical acts have been authorised for pharmacists: vaccination, prescription review, monitoring of anti-vitamin K treatments, for example. Several qualitative studies have examined the experiences and perceptions of health professionals regarding interprofessional cooperation. In these studies, several factors for optimal management of patients with polypharmacy have been proposed: interprofessional cooperation, including with pharmacists, a partnership relationship with specialists, with greater transparency and equality [13,14]. However, little is known about how GPs cooperate with other health professionals (specialist doctors and non-medical professionals such as pharmacists, nurses, physiotherapists etc) and to what extent this cooperation takes place in the management of patients with multimorbidity and polypharmacy.

The objectives of our study were: (1) to identify different profiles of GPs, according to their opinions and attitudes towards cooperation with other health professionals in managing patients with multimorbidity and their prescriptions (2) to study the personal and professional characteristics of GPs associated with these profiles.

## Methods

### Design and population

We conducted a cross-sectional survey on managing patients with multimorbidity and polypharmacy, among a panel of GPs in private practice in France. The panel design has been described in previous publications [15]. In brief, we randomly selected GPs from the exhaustive French database of health professionals (Répertoire Partagé des Professionnels de Santé) between December 2013 and March 2014. Sampling was stratified on age, gender, the annual number of consultations and home visits, and the medical density of each GP’s municipality of practice. We excluded GPs with fewer than five patient consultations per week, those planning to retire within six months, and those practising exclusively alternative medicine (e.g. acupuncture or homoeopathy).

### Data collection procedure and questionnaire

Upon inclusion in the panel, participants answered a short questionnaire about their professional characteristics and then on the organisation of their practice: solo practice, group practice (several doctors practising together), and multi-professional practice (GPs working with several health professionals including nurses, physiotherapists, psychologists, etc). Using a standardised questionnaire, professional interviewers used a computer-assisted telephone interview system to collect data between May and July 2016. The questionnaire was based on a literature review and the results of two focus groups, one of four and one of five GPs. It was developed with a multidisciplinary group of GPs with expertise in multimorbidity, pharmacologists, epidemiologists et statisticians. We pilot-tested the questionnaire with 50 GPs to clarify and validate the questions.

We defined multimorbidity as the presence of several chronic diseases in one person. Polypharmacy was illustrated by an example in a case vignette of a woman with several chronic diseases and taking several medications. The questionnaire asked GPs about their opinions and their role and that of specialists in managing prescriptions for patients with multimorbidity and their opinions of their cooperation with pharmacists in polypharmacy (4-point Likert scale ranging from strongly disagree to strongly agree, see Additional File). We asked GPs about the frequency of contacts with specialists or pharmacists for medication management (4-point Likert-like scale: never, sometimes, often and very often). We asked them about the usefulness of the participation of non-physician health professionals such as nurses in managing these patients, through their consultations or interprofessional meetings (Yes/No). A ‘don’t know’ response was also provided for each question.

### Statistical analysis

Data were weighted to match the national GP population in 2016 according to the stratification variables, despite the French GP population’s attrition and changes between inclusion and data collection. For objective 1, multiple correspondence analysis (MCA) was used as a pre-processing step for further cluster analysis: this allows us to reduce the data’s dimensionality and transform categorical variables into continuous variables (factorial coordinates). Then we conducted an agglomerative hierarchical cluster analysis (AHCA) to identify cooperation clusters (profiles) of GPs, according to their opinions and attitudes regarding cooperation with other health professionals (the Additional file contains the items included in the analysis), using the Ward linkage method with squared Euclidean distance measure. We have performed a robustness check to ensure that the results remain when the items are treated as continuous (rather than categorical) variables, using the AHCA directly (without prior use of MCA). While the number of individuals in each cluster was slightly different (*n* = 259 for ‘Very favourable to collaboration’ profile; 364 for ‘Moderately favourable to collaboration’ profile; 144 for ‘Selectively favourable to collaboration’ profile’ and 336 for ‘Not favourable to collaboration profile’, we can distinguish the same profiles as before. We used the minimum lost inertia to identify the optimal number of clusters; this corresponds to a minimal intra-cluster variance (individuals with maximum similarity in each cluster) and a maximal inter-cluster variance. We have also used semi-partial *R*-square and *F*-statistic and pseudo-*t*^2^ criteria. To perform the MCA, for items that had categories accounting for less than 10% of the answers, these categories were grouped with the closest category (e.g. often/very often; see Additional file). Any individual with at least one non-response or ‘do not know’ answer was excluded from the analysis.

For objective 2, we used the cooperation clusters as dependent variables and performed multinomial logistic regressions to study their associations with GPs’ personal and professional characteristics (age, gender, practice organisation and participation in continuing medical education (CME)). Logistic regressions were adjusted for workload, medical density and the reported proportion of patients with multimorbidity on GP’s list. All analyses were based on two-sided *P* values, with statistical significance defined as *p* ≤ .05 and were performed with SAS 9.4 statistical software (SAS Institute, Cary, NC).

### Ethics

Consent to participate was obtained from each general practitioner at the time of the inclusion in the panel. The National Authority for Statistical Information (Commission Nationale de l'Information Statistique) approved the panel and its surveys (Paris, June the 3rd 2013, n°82/H030). This national institution evaluated that the study was in accordance with the rules and regulations regarding the protection of personal data.

## Results

Of the 3,724 eligible GPs, 1,712 (46.0%) agreed to join the panel in 2014, of which 1,266 (73.9%) were still participating at the time of this survey in 2016. Of these, 1,183 (31.8% of eligible GPs) completed the multimorbidity/polypharmacy questionnaire. In addition, individuals that had at least one non-response or ‘do not know’ answer (*n* = 81) were excluded from the analysis. Table 1 describes the characteristics of the participants. The excluded GPs were not significantly different from the respondents in terms of age, gender, the annual number of consultations and house calls, and the medical density of each GP’s municipality of practice. Thus, the MCA used 12 variables with 34 categories (detailed MCA results available on request).

**Table 1.** Characteristics of the sample from the national panel of GPs, France, May-September 2016 (descriptive analyses of weighted data), *N* = 1,183.

	*n*	Frequency, %
**Stratification variables**		
***Age at inclusion in years (tertiles)***		
<50	362	30.6
50–58	386	32.6
>58	435	36.8
***Gender***		
Women	363	30.7
***Workload (number of consultations/visits from December 2011 to November 2012)***		
< 3067	294	24.8
3067–6028	592	50.1
> 6028	297	25.1
***GP density of the municipality of practice in 2012***		
< −19.3% of the national average	296	25.0
−19.3% to + 17.7% of the national average	591	50.0
> + 17.7% of the national average	296	25.0
**Professional characteristics**		
***Practice organisation in 2016*** *		
Solo practice	537	49.4
Group practice^a^	482	44.3
Multi-professional practice^b^	69	6.3
***Participated in a continuing medical education (CME) course in 2012***†		
No	133	11.2
Yes	996	84.2
***Self-reported proportion of patients with multimorbidity on GP’s list***‡		
< 25%	501	42.9
25%–50%	497	42.5
> 50%	170	14.6

GP = general practitioner. The sample was representative of the French private practice GPs population for stratification variables in 2016 (Sampling weights).

*Data from the third survey of the national panel, 95 missing data.

^a^Practice organisation where many physicians work together.

^b^Practice organisation where GPs work with several health professionals (nurses, physical therapists, psychologists, specialists, etc).

†54 missing data.

‡15 ‘no answer’.

### GPs' profiles on interprofessional cooperation

Cluster analysis identified four profiles of GPs, according to their opinions on the roles of different professionals in the management of patients with multimorbidity and polypharmacy and their attitudes and opinions on cooperation with these professionals (Table 2).

**Table 2.** Profiles of GPs according to their opinions and behaviours about cooperation with health professionals for management of patients with multimorbidity and polypharmacy, *N* = 1,102*.

	Very favourable to cooperation profile	Moderately favourable to cooperation profile	Selectively favourable to cooperation profile	Non-cooperative profile	All	*P* Value
	*N* = 158 (14%) Frequency, %	*N* = 511 (47%) Frequency, %	*N* = 300 (27%) Frequency, %	*N* = 133 (12%) Frequency, %	Frequency, %	
**Age at inclusion in years (tertiles)**						
<50	26.0	36.5	31.7	21.8	31.7	.004
50–58	32.8	33.4	28.4	33.3	31.9	
> 58	41.3	30.1	40.0	45.0	36.4	
**Women**	26.3	33.4	30.7	22.6	30.2	.07
**Practice organisation in 2016**						
Solo practice	56.8	40.3	46.9	43.3	44.9	.004
Group practice	34.9	51.9	48.1	54.7	48.8	
Multi-professional practice	8.4	7.8	5.0	1.9	6.4	
**Participated in a continuing medical education (CME) course in 2012**						
Yes	90.2	91.7	85.4	82.2	88.6	.009
**GP's opinion about their role and that of specialists in the management of prescriptions**						
***You are the one who decides the prescriptions, even for medications initially prescribed by another physician***						
Agree/Strongly agree	84.2	75.3	80.9	76.4	78.3	.09
***You feel you are well informed about all medications taken by your patients with multimorbidity***						
Agree/Strongly agree	83.1	76.1	93.5	86.9	83.3	<.001
***Specialists are well informed about all medications taken by their patients***						
Agree/Strongly agree	58.0	40.9	67.6	37.8	50.3	<.001
***Management of patients with multimorbidity by different specialists increases the risk of drug interactions***						
Strongly disagree/Disagree	12.9	4.5	38.2	8.2	15.3	<.001
**GPs' opinions about pharmacists' role and cooperation with them**						
***The pharmacist is the professional who knows all patients' medications best***						
Agree/Strongly agree	91.7	73.5	56.1	70.8	71.2	<.001
***GPs and pharmacists don't collaborate enough on patients' polypharmacy***						
Agree/Strongly agree	54.3	75.3	38.7	69.2	61.4	<.001
***You expect the pharmacist to warn you of drug-interaction risks among a patient's prescriptions***						
Agree/Strongly agree	95.2	93.5	87.2	90.7	91.7	<.001
**GPs' behaviours about the cooperation with various health professionals**						
***Usefulness of meeting with patients' other health professionals***						
Yes	70.7	80.9	42.2	46.5	64.4	<.001
***To learn what medications have been prescribed to a patient, you call the pharmacist***						
Often/very often	61.4	60.1	46.0	25.8	52.1	<.001
***To learn what medications have been prescribed to a patient, you call the physician who prescribed them***						
Often/very often	48.5	48.2	50.4	26.9	46.1	<.001
**GPs' opinions about professional delegation**						
***The pharmacist has enough information to modify patient's medications***						
Agree/Strongly agree	71.2	2.7	0.5	5.8	12.9	<.001
***Usefulness of consultations by nurses for patients with chronic diseases***						
Yes	72.3	69.8	43.9	42.4	59.6	<.001

Agglomerative hierarchical cluster analysis, France, May-September 2016 (*N* = 1,102).

*Participants with at least one non-response or ‘do not know’ response (*N* = 81) were excluded from the analysis. The sample remained representative of the population of French private practice GPs for stratification variables in 2016 (Sampling weights).

GPs from the ‘very favourable to cooperation’ profile (14% of the sample) had a favourable opinion of the role of specialists and other health professionals in the management of this patient population and their cooperation with them. Only this group had a majority (71%) of agreement with the delegation to pharmacists of the medicines’ management (review and modification of the prescription). GPs from the ‘moderately favourable to cooperation’ profile (47%) had more positive opinions than average on the knowledge and role of pharmacists in managing polypharmacy, but they were mostly opposed to pharmacists changing patients’ prescriptions. They were more favourable than average to cooperation with different health professionals in the management of these patients, including delegation of consultations to nurses but had more negative opinions than average on the role of specialists in this management. GPs in the ‘selectively favourable to cooperation’ profile (27%) were likely to cooperate with other doctors and had more positive views than average on the role of specialists in managing medicines. They tended to oppose the delegation of prescriptions to nurses or pharmacists and had unfavourable opinions on the role and cooperation of pharmacists. GPs from the ‘non-cooperative’ profile (12%) expressed negative views on cooperation, the delegation of tasks, and the role of other professionals in polypharmacy management.

### Associations between GPs' profiles and their characteristic

Multinomial logistic regression (Table 3) with the ‘non-cooperative’ profile as the reference showed that GPs with a ‘moderately favourable to cooperation’ profile was the youngest. The latter, as well as those with a ‘very favourable to cooperation' profile, were also more likely to have participated in CME. GPs' profiles were not associated with their workload, medical density, or self-reported proportions of patients with multimorbidity in their practice. They were not associated with practice in multi-professional groups, but those with a ‘very favourable to cooperation’ profile worked less frequently in group practices. Overall, these results indicate that differences between the three ‘cooperative’ profiles are smaller than differences with the ‘non-cooperative’ reference profile.

**Table 3.** Association between GPs' profiles according to their opinions and behaviours about cooperation and personal and professional characteristics.

	GPs' profiles (ref. Non-cooperative profile)					
	Very favourable to cooperation profile		Moderately favourable to cooperation profile		Selectively favourable to cooperation profile	
Characteristics	aOR* (95% CI)	*P* Value	aOR* (95% CI)	*P* Value	aOR* (95% CI)	*P* Value
***Age at inclusion in years (ref. <50)***						
50–58	0.79 (0.42–1.48)	0.46	0.63 (0.38–1.04)	0.07	0.74 (0.43–1.28)	0.28
> 58	0.78 (0.39–1.56)	0.48	**0.45 (0.25–0.80)**	**0.007**	0.75 (0.40–1.37)	0.35
***Gender (ref. Men)***						
Women	1.14 (0.63–2.06)	0.67	1.56 (0.96–2.53)	0.07	1.44 (0.85–2.42)	0.17
***Practice organisation in 2016***† ***(ref. Solo practice)***						
Group practice^a^	**0.52 (0.30–0.90)**	**0.02**	0.77 (0.49–1.20)	0.24	0.72 (0.45–1.16)	0.18
Multi-professional practice^b^	2.09 (0.54–8.14)	0.29	2.98 (0.87–10.25)	0.08	1.78 (0.48–6.58)	0.38
***Participated in a continuing medical education course in 2012 (ref. No)***						
Yes	**2.09 (1.00–4.37)**	**0.05**	**2.77 (1.52–5.03)**	**<.001**	1.32 (0.73–2.39)	0.36

GP = general practitioner, aOR = adjusted odds ratio, CI = confidence interval.

*Multinomial logistic regressions (*N* = 953, non-weighted data) adjusted for workload, medical density, reported proportion of patients with multimorbidity on GP’s list.

†Data from the third survey of the national panel.

^a^Practice organisation where many physicians work together.

^b^Practice organisation where GPs work with several health professionals (nurses, physical therapists, psychologists, specialists, etc).

Bold values correspond to significant results.

## Discussion

### Main findings

The 1,183 GPs who responded to the questionnaire were representative of the population of French GPs according to age, gender, the annual number of consultations and home visits, and medical density of their municipality of practice. We found different profiles of GPs' attitudes towards cooperation and sharing the management of prescriptions for patients with multimorbidity and polypharmacy, with specialists and other health professionals. The most represented profile was ‘moderately favourable’, where GPs favoured cooperation with specialists and primary care professionals, without going so far as to delegate prescription review to pharmacists. GPs belonging to the most cooperative profiles (moderately favourable and very favourable) had attended more CME courses than those in the ‘non-cooperative’ profile.

#### Comparison with literature

##### GPs and specialists: a complex cooperation

Previous qualitative studies have shown that many GPs, in different countries, feel that the involvement of many specialists in the management of patients with multimorbidity leads to fragmentation of care and increase polypharmacy and treatment burden, mainly due to a lack of communication between doctors [4,13]. In these studies, many GPs call for better interprofessional communication and a fair balance between them and specialists when sharing prescribing activity [13,14]. Our quantitative study confirmed that cooperation and trust between GPs and specialists regarding prescription management are not evident for most GPs (‘moderately favourable’ and ‘non-cooperative’ profiles).

##### GPs-nurses cooperation: effective and popular

General practitioners prefer to transfer patient counselling (lifestyle, disease monitoring) and therapeutic education to nurses and sometimes pharmacists for patients with chronic diseases [16]. Our finding that most GPs favoured nurse practitioners providing consultations for patients with multimorbidity is encouraging. Literature reviews on interprofessional cooperation have shown that nurse practitioners’ involvement in the follow-up of these patients achieved equal or better outcomes for chronic patients than primary care doctors in terms of quality of care, health status, and patient satisfaction [10,17]. But a key element for effective cooperation is structured communication between GPs and nurse practitioners [18].

##### The challenges of GPs-pharmacist cooperation

Our results highlight the diversity of GPs' views on the role of pharmacists in managing polypharmacy. A minority of GPs favoured delegating prescription review to the pharmacist. However, a Belgian study showed that pharmacist prescription review could be accepted by two-thirds of GPs when explained in a face-to-face pharmacist-GP discussion [19]. Previous studies have shown that pharmacists’ involvement in medication management can lead to better clinical outcomes, improved prescribing habits and better patient quality of life, particularly for patients with polypharmacy [20,21]. Their involvement inpatient counselling, therapeutic education or training of other health professionals positively impacts patient's follow-up, adherence and quality of life [20]. GP-pharmacist cooperation is often limited to clarifying prescriptions, information about medicines or patient history [22,23]. Contacts are occasional and initiated mainly by the pharmacist. Nevertheless, pharmacists and GPs agree that cooperation is easier when they have a local and long-standing working relationship [22,24].

##### What are the elements associated with greater cooperation?

GPs with a ‘moderately favourable to cooperation’ profile, were younger than others. This may reflect the shaping of attitudes towards interprofessional cooperation by years of experience and professional environment: more experienced GPs may have stronger views on the role of other health professionals in their area.

The lack of association between GPs’ profiles and the organisation of multi-professional practice is surprising. However, our study was mainly interested in the roles of specialists and pharmacists, who are rarely integrated into such organisations in France. Multi-professional and group practices themselves have varied profiles of interprofessional organisation and collaboration [25].

Our results indicate that most cooperative GPs have an interest in continuing education. The inclusion of multi-professional courses in the training programs for medical, pharmacy, nursing, and other health students can lead to a better understanding of the roles and competencies of each profession and foster their future cooperation [6,26].

### Strengths and limitations

Our sample size was large. In addition, we weighted the data according to some characteristics of GPs to minimise selection bias. The AHCA method allowed us to study the statistical proximity of individuals based on the factors studied, without any preconceived notion of possible relationships between these factors. This analysis allowed us to identify clusters of GPs evaluated for several factors.

Among the limitations, the data collected was self-reported. Thus, social desirability or conformity biases cannot be excluded. The use of Lickert scales and closed-ended responses meant that GPs responded to their opinions and attitudes in general and not to the wide variety of situations they encounter in their practice. We could not measure real cooperation practices in various multimorbid patient situations. Such a study would have required a more complex design. The data was collected and weighted in 2016, so the characteristics of French GPs may have evolved by 2021.

### Practice and policy implications

Health policies in different countries encourage interprofessional cooperation to improve the quality of primary care and patient health [10,12,27], especially for multimorbidity patients [28]. Our study highlighted the diversity of GPs' views on cooperation with other health professionals and sharing the management of prescriptions. While most GPs favoured interprofessional cooperation, they are also reluctant primarily to delegate prescribing tasks. Policymakers need to be aware of these disparities and difficulties.

Better communication and understanding of each other's roles, knowledge and responsibilities are essential for improved cooperation [13,18,24]. The rapid development of multi-professional medical centres in France and elsewhere is an opportunity to stimulate and facilitate this cooperation.

## Conclusion

There are disparities between GPs regarding interprofessional cooperation in managing patients with multimorbidity and polypharmacy. Most GPs are willing to cooperate with other primary care professionals and specialists. Some GPs prefer to cooperate with other doctors and only a minority are willing to share treatment management with non-physician professionals. Interprofessional education, whether for students or professionals, could be a way to improve knowledge and understanding of each professional's roles and skills in the management of complex patients.

Availability of data and materials: The datasets used and analysed during the current study are available from the corresponding author on reasonable request. Descriptive analysis of data used in the current study is shown in an additional file.

## Supplementary Material

Supplementary Data 2

Supplementary Data 1
