# Supplementary material for: General practitioners' attitude towards cooperation with other health professionals in managing patients with multimorbidity and polypharmacy: A cross-sectional study
Source: Eur J Gen Pract. 2022 May 20;28(1):109–17. doi: 10.1080/13814788.2022.2044781 (PMC9132456; doi:10.1080/13814788.2022.2044781)
Supplement: Supplementary Data 2 [file IGEN_A_2044781_SM1108.docx]

**Supplementary data 2. Results of the AHCA classification, intra- and intercluster variance (n=1,102)**

| **Class** | | | **1** | **2** | **3** | **4** | **5** | **6** |
| --- | --- | --- | --- | --- | --- | --- | --- | --- |
| **6 classes** | **Intercluster variance** | 0.2483 | 0.0306 | 0.0428 | 0.0476 | 0.0437 | 0.0325 | 0.0511 |
|  | **Intracluster variance** | 0.7120 | 0.0844 | 0.2222 | 0.1232 | 0.0781 | 0.1008 | 0.1033 |
| **5 classes** | **Intercluster variance** | 0.2174 | 0.0322 | 0.0428 | 0.0476 | 0.0437 | 0.0511 | - |
|  | **Intracluster variance** | 0.7429 | 0.2161 | 0.2222 | 0.1232 | 0.0781 | 0.1033 | - |
| **4 classes** | **Intercluster variance** | 0.1740 | 0.0324 | 0.0428 | 0.0476 | 0.0511 | - | - |
|  | **Intracluster variance** | 0.7863 | 0.3376 | 0.2222 | 0.1232 | 0.1033 | - | - |
| **3 classes** | **Intercluster variance** | 0.1203 | 0.0324 | 0.0402 | 0.0476 | - | - | - |
|  | **Intracluster variance** | 0.8400 | 0.3376 | 0.3792 | 0.1232 | - | - | - |
| **2 classes** | **Intercluster variance** | 0.0663 | 0.0260 | 0.0402 | - | - | - | - |
|  | **Intracluster variance** | 0.8940 | 0.5148 | 0.3792 | - | - | - | - |
| **Total inertia** | | 0.9603 |  |  |  |  |  |  |
