# Supplementary material for: General practitioners' attitude towards cooperation with other health professionals in managing patients with multimorbidity and polypharmacy: A cross-sectional study
Source: Eur J Gen Pract. 2022 May 20;28(1):109–17. doi: 10.1080/13814788.2022.2044781 (PMC9132456; doi:10.1080/13814788.2022.2044781)
Supplement: Supplementary Data 1 [file IGEN_A_2044781_SM1107.docx]

| **Supplementary data 1. GPs' opinions and attitudes about collaboration with health professionals for management of patients with multimorbidity and polypharmacy, France, May-September 2016 (N = 1102*, descriptive analyses of weighted data)** | | | | |  |
| --- | --- | --- | --- | --- | --- |
| **GP's opinion about their role and that of specialists in the management of prescriptions** | **Strongly disagree**  **(%)** | **Disagree**  **(%)** | **Agree**  **(%)** | **Strongly agree**  **(%)** | |
| You are the one who decides the prescriptions, even for medications initially prescribed by another physician | 3.2 | 18.5 | 50.6 | 27.7 | |
| You feel you are well informed about all medications taken by your patients with multimorbidity | 1.2 | 15.6 | 58.4 | 24.8 | |
| Specialists are well informed about all medications taken by their patients | 9.4 | 40.3 | 40.8 | 9.5 | |
| Management of patients with multimorbidity by different specialists increases the risk of drug interactions | 4.4 | 10.9 | 44.6 | 40.1 | |
| **GPs' opinions and attitudes about the collaboration with various health professionals** | | | | |  |
| The pharmacist is the professional who knows all patients' medications best | 7.4 | 21.4 | 52.7 | 18.5 | |
| The pharmacist has enough information to modify patients' medications | 44.8 | 42.3 | 11.0 | 1.9 | |
| GPs and pharmacists don't collaborate enough on patients' polypharmacy | 8.3 | 30.2 | 42.7 | 18.8 | |
| You expect the pharmacist to warn you of drug-interaction risks among a patient's prescriptions | 2.7 | 5.6 | 37.9 | 53.8 | |
| **GPs’ attitudes to learn what medications have been prescribed** | **Never** | **Sometimes** | **Often** | **Very often** | |
| To learn what medications have been prescribed to a patient, you call the pharmacist | 11.7 | 36.2 | 28.9 | 23.2 | |
| To learn what medications have been prescribe to a patient, you call the physician who prescribed them | 10.5 | 43.4 | 25.6 | 20.5 | |
| **GPs’ opinions about other health professionals’ interventions** | **No** | | **Yes** | |  |
| Usefulness of meeting with patients' other health professionals | 35.6 | | 64.4 | |  |
| Usefulness of consultations by nurses for patients with chronic diseases | 40.4 | | 59.6 | |  |

^a^ Participants that presented at least one non-response or ‘do not know’ answer (n=81) were excluded from the analysis. The sample remained representative of French private practice GPs population for stratification variables, in 2016.
